# Rabies in marmosets (Callithrix jacchus), Ceará, Brazil.

**DOI:** 10.3201/eid0706.010630

**Published:** 2001

**Authors:** S R Favoretto, C C de Mattos, N B Morais, F A Alves Araújo, C A de Mattos

**Affiliations:** Instituto Pasteur São Paulo, Brazil.

## Abstract

A new Rabies virus variant, with no close antigenic or genetic relationship to any known rabies variants found in bats or terrestrial mammals in the Americas, was identified in association with human rabies cases reported from the state of Ceará, Brazil, from 1991 to 1998. The marmoset, Callithrix jacchus acchus, was determined to be the source of exposure.


### Rabies in Marmosets (*Callithrix jacchus*), Ceará, Brazil

**Silvana R. Favoretto,* Cecilia C. de Mattos,† Nelio B. Morais,‡ Francisco A. Alves Araújo,§ and Carlos A. de Mattos†**

*Instituto Pasteur São Paulo, São Paulo, Brazil; †Centers for Disease Control and Prevention, Atlanta, Georgia, USA; ‡Secretaría Estadual de Saúde do Ceará, Brazil; and §Ministerio da Saúde, Brasilia, Brazil

A new *Rabies virus* variant, with no close antigenic or genetic relationship to any known rabies variants found in bats or terrestrial mammals in the Americas, was identified in association with human rabies cases reported from the state of Ceará, Brazil, from 1991 to 1998. The marmoset, *Callithrix jacchus jacchus*, was determined to be the source of exposure.

Canine rabies has been controlled in southern Brazil, and cases have declined in the rest of the country (1). Under these new epidemiologic conditions, the existence of rabies in other species, until now eclipsed by the rabies cycle in dogs, has become evident (2-4).

In the state of Ceará, for example, 13 human deaths due to rabies transmitted by wildlife were reported from 1991 to 1998 (Figure 1). Surveillance data indicated that these deaths were the consequence of exposure to bats (four deaths), a crab-eating raccoon (*Procyon cancrivorous* [guaximín]; one death), and the white-tufted-ear marmoset (*Callithrix jacchus jacchus* [sagüí]; eight deaths). These last eight cases constitute the first report in which one species of the order Primate is a primary source of rabies infection for humans in a restricted geographic area.

The marmoset, *C. j. jacchus* or sagüí, is a small diurnal primate that feeds on insects, fruits, and tree exudates (5). Members of this species are commonly captured to keep as pets in Ceará, as other marmosets are in the rest of the country (6) (Figure 2). The sagüí is also present in the neighboring states of Piauí and Pernambuco (Figure 1) (5). These marmosets are highly adaptable to different habitats and can be found on plantations and in urban parks (5,7).

In one of the eight human rabies cases associated with *C. jacchus*, the animal approached the house and attacked the owner. In another case, the marmoset was raised as a pet. In the other cases, the exposure occurred during attempts to capture the animals (4). Seven of these cases occurred in the coastal region, where the sagüí is more abundant (4). Epidemiologic investigations and surveillance data suggested the emergence of a rabies cycle in which this marmoset was the main transmitter. The public health importance of this situation is reflected not only in reported human deaths, but also in the fact that in Ceará an average of 25 persons per month seek rabies postexposure prophylaxis for marmoset and other primate bites (4).

To better understand the underlying factors that could be responsible for the emergence of these unusual epidemiologic events and to help identify a possible reservoir of this *Rabies virus* (RABV) circulating in Ceará, three rabies field isolates—two obtained from humans, each bitten by a different sagüí (Brhm4097 and Brhm4108), and one from a rabid sagüí (Brsg4138) in 1998—were antigenically and genetically characterized.

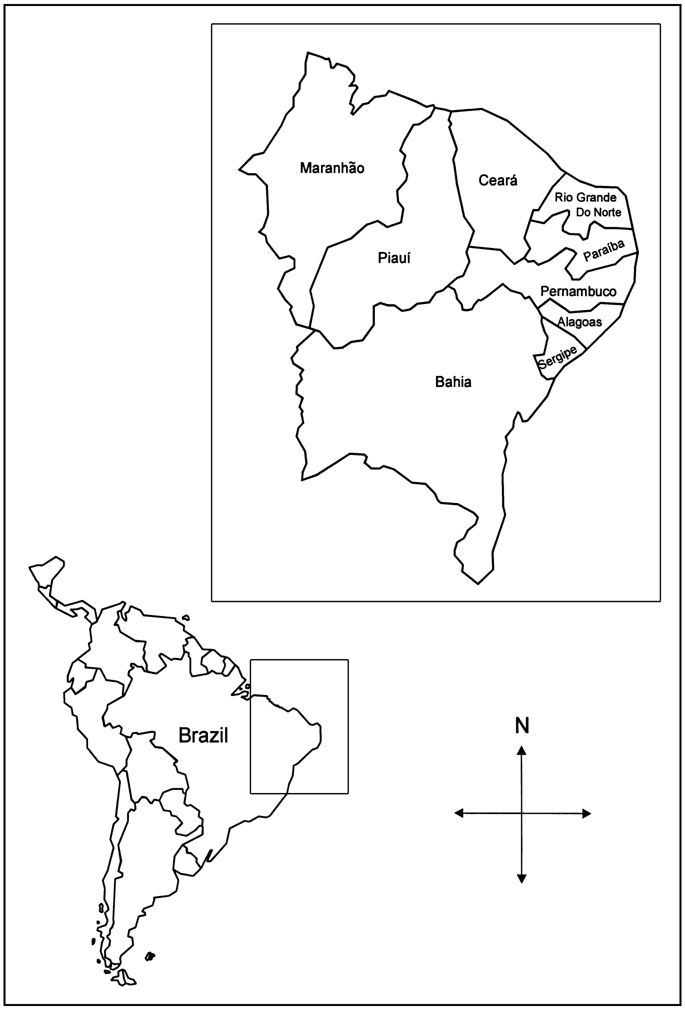

Figure 1. Map of Brazil indicating the location of Ceará State.

Address for correspondence: Cecilia C. de Mattos, Viral and Rickettsial Zoonosis Branch, Division of Viral and Rickettsial Diseases, Centers for Disease Control and Prevention, 1600 Clifton Road, Mailstop G33, Atlanta, Georgia 30333, USA; fax: 404-639-1058; e-mail: cdd8@cdc.gov

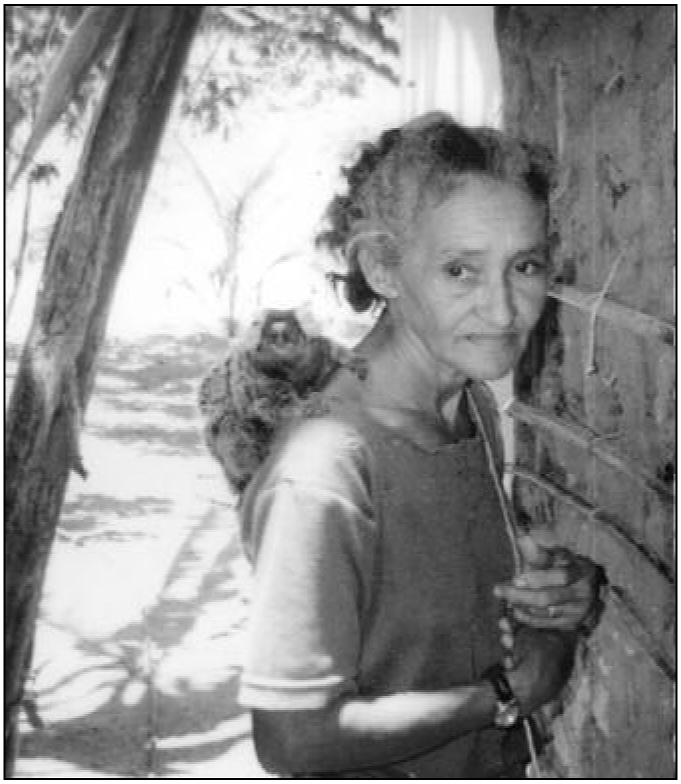

Figure 2. A pet marmoset with its owner.

### The Study

Case Brhm4097 was in a 31-year-old woman who died with a clinical diagnosis of rabies, later confirmed by a direct immunofluorescence-antibody (DIF) test. Interviews with the patient and her relatives indicated that she had been attacked and bitten on the leg by a sagüí near her house. Case Brhm4108 was in a 17-year-old woman who was hospitalized with a presumptive case of meningitis. Upon evaluation, a clinical diagnosis of rabies was made and later confirmed with a DIF test. Interviews with the patient and family members revealed that she had been bitten on one ear by a sagüí captured in the forest 4 days earlier. In both cases, those bitten did not request postexposure prophylaxis because they were not aware of any potential risk for rabies transmission by this animal. Case Brsg4138 was a *C. j. jacchus* kept as a pet, which was brought to health authorities because it exhibited behavioral changes. This marmoset was euthanized and sent to the laboratory to be tested for rabies; RABV antigen was identified in its brain with a DIF test.

These three isolates were compared with RABV variants circulating among dogs in Latin America, with other RABV variants maintained by sylvatic terrestrial reservoirs in the United States (north-central skunk, south-central skunk [*Mephitis mephitis*], Arizona and Texas fox [*Urocyon cinereoargenteus*], California skunk [*M. mephitis*], Texas coyote [*Canis latrans*], and raccoon [*Procyon lotor*]) and with isolates obtained from terrestrial wildlife in Latin America in which the reservoirs were either unknown, e.g., a fox in Peru and a cat in Venezuela, or unconfirmed, e.g., skunks in Mexico. The three Brazilian viruses were further analyzed and compared with RABV from endemic cycles maintained in migratory bats (red bat [*Lasiurus cinereus]*, free-tailed bats [*Tadarida brasiliensis*], nonmigratory insectivorous bats (big brown bat [*Eptesicus fuscus]*, and California myotis [*Myotis californicus*]) in the United States. South American RABV isolates obtained from vampire bats (*Desmodus rotundus*), vampire bat-related cases, and insectivorous bat species that are known rabies reservoirs (lasiurine species and free-tailed bats) or unconfirmed reservoirs were also included in this comparison (8-12).

Antigenic characterization was conducted with a panel of eight monoclonal antibodies (MAbs) elicited against the viral nucleoprotein (8). Primary isolations were made by mouse intracranial inoculation. Acetone-fixed touch impressions of infected mouse brain material were reacted with the MAbs by indirect immunofluorescence techniques (13,14).

Antigenic characterization of the three isolates revealed only one reaction pattern. The isolates reacted positively with MAb C9 and C10 and negatively with the other six MAbs. Comparison of this reaction pattern with those that characterize RABV variants in known reservoirs in the Americas (13,14) indicated that the antigenic profile of these three viruses has not been previously identified.

Genetic analysis of the samples was performed by sequencing part of the nucleoprotein gene (from position 1,157 to position 1,476, as compared with RABV SADB19 [15]), then comparing its phylogenetic relationship with the RABV variants described above (8,12).

For the sequencing studies, viral RNA was extracted from the infected tissues with TRIzol (Invitrogen, San Diego, CA). The cDNA was obtained by reverse transcription-polymerase chain reaction techniques by using primers 10g and 304, as described (8,16). The cDNA was sequenced with primer 304 by using the Taq Big Dye Terminator Cycle Sequencing Ready Reaction Kit (Applied Biosystems Inc., Foster City, CA) on an Applied Biosystems 377 DNA automated sequencer (Applied Biosystems), as reported (8).

For phylogenetic analyses, the PileUp program of the Wisconsin Package Version 10.1 (Genetic Computer Group, Madison, WI) (17) was used to construct the alignment of viral sequences. Further analyses were carried out with distance matrix methods, as implemented in the PHYLIP Package, Version 3.5 (18), using the programs DNADIST (Kimura-two parameter method) and NEIGHBOR (neighbor-joining method). The confidence value for each node (100 replicates) was assessed with the SEQBOOT program, and the consensus tree was obtained with the CONSENSE program of the same package. Trees were constructed with the TREEVIEW program (19).

Comparative phylogenetic analyses by the distance matrix methods yielded a tree in which all viruses segregated into two main groups, identified as A (bootstrap value 57%) and B (bootstrap value 99%) (Figure 3). The Ceará viruses were segregated in group A, which included RABV genetic variants circulating in hematophagous and nonhematophagous bats in the Americas and viruses of raccoon and south-central skunk cycles in the United States. The topologic association between RABV genetic variants circulating in well-recognized terrestrial reservoirs and variants maintained by bat species has been previously described (20). Group B was formed by viruses circulating in dogs and terrestrial wildlife in the Americas.

In group A the Brazilian isolates clustered together, forming a discrete clade A1 (bootstrap 100%) demonstrating their distinct nature (Figure 3). Viruses Brsg4108 and

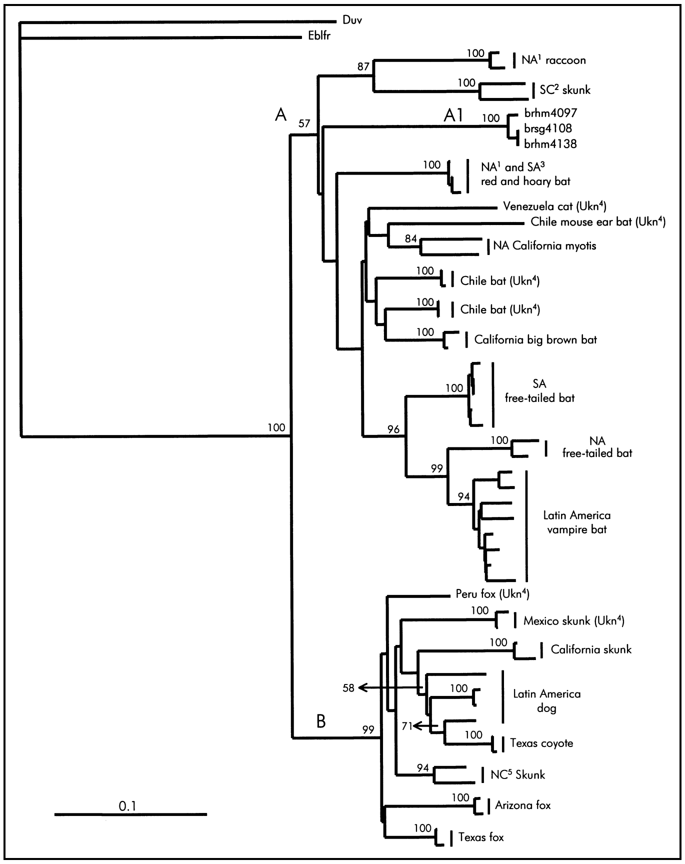

Figure 3. Neighbor-joining tree of the comparison of the Ceará samples with isolates obtained from domestic and wild animals from the Americas. Bootstrap values obtained from 100 resamplings of the data using distance matrix methods are shown at the corresponding nodes. Only bootstrap values >50% are shown at branching points. Bar in left corner indicates 0.1 nucleotide substitutions per site. Significance of letter designations at the nodes are discussed in the text. a: North America; b: south central USA; c: South America; d: unknown reservoir; e: north central USA.

Brhm4138 showed no nucleotide difference between them and revealed a genetic distance value of only 1.3% with sample Brhm4097. The remarkable genetic relatedness of the group A1 members was in clear contrast to the high percentage of genetic distance that they showed with all other rabies variants. The genetic distances between Group A1 and all other rabies variants ranged from 20% (lasiurine species) to 34% (skunks from Mexico). Although bootstrap analyses highly supported terminal nodes that clearly defined each of the rabies genetic variants in group A, as in group B this method did not render significant values in most of the intermediate nodes; thus, no genetic relationships between the Ceará viruses and the other variants could be determined.

### Conclusions

The high degree of genetic homology among the Ceará rabies viruses, in conjunction with surveillance data, demonstrated an epidemiologic linkage between the viruses and confirmed that they represent a unique and independent rabies endemic cycle.

These findings are also noteworthy because nonhuman primates have rarely been reported rabid in the wild, and they have been involved only sporadically in cases of human exposures to RABV (21-23). Rabies affects wild and domestic animals as well as humans. Minor changes in any of these populations or in their environment can result in the emergence or reemergence of the disease in a geographic area (24). Several probable factors could have caused the emergence of rabies in the *C. j. jacchus* population of Ceará. First, small changes produced by human activities are not necessarily deleterious for survival of *C. j. jacchus*. Indeed, such modifications might be beneficial for this species in the long term and could contribute to an increase in its population (6). The proximity of this marmoset to urban settlements and the common practice of capturing and keeping them as pets are two other contributing factors. Additionally, both the reduction in rabies incidence among dogs in the state (1,2) and the improvement of rabies surveillance programs by incorporating the molecular characterization of rabies isolates contributed to the recognition of this cycle in Ceará.

Wildlife unable to support rabies endemic cycles are sporadically infected (spillover) as a result of their interaction with rabid animals from sympatric species capable of perpetuating the virus in nature (reservoirs). The infection of *C. j. jacchus* with this distinct RABV genetic variant could be the result of spillover from an unknown reservoir. Until now, available data did not provide enough evidence to determine the nature (Chiroptera vs. Carnivora) of this putative reservoir. Although mostly arboreal, *C. j. jacchus* descends to the ground to feed on insects or to cross clearings in the forest, behaviors that may expose them to bats or terrestrial reservoirs. Alternatively, these events could be evidence of *C. j. jacchus* acting as a rabies reservoir. To act as a rabies reservoir, a species should be highly susceptible to the virus, undergo variable incubation periods, excrete the virus in saliva at the appropriate concentration to infect conspecifics, and maintain a high population density and turnover (25).

Field studies of *Callithrix* spp. are limited (7), but some characteristics of the natural history of this species correspond to those of a typical rabies reservoir. Callitrichids are some of the most successful species in regard to geographic distribution, population density, and habitat exploitation (26). A population of 700 animals per square kilometer was observed in a 3-Ha forest fragment in Brazil (27). Although as yet no experimental inoculation studies mimicking the natural route of infection have been conducted to investigate the pathobiology of rabies in *C. j. jacchus*, this animal is highly susceptible to the intracerebral inoculation of the virus (28). Improvement in basic surveillance programs for rabies in wildlife, public education, and determination of rabies seroprevalence in *C. j. jacchus* in Ceará would help elucidate this new epidemiologic puzzle.

### Acknowledgments

We thank Charles E. Rupprecht and staff for their collaboration, advice, and valuable discussions during the preparation of this manuscript. The Centers for Disease Control and Prevention produced and provided the monoclonal antibodies used in this study.

Dr. Favoretto is a specialist in public health at the Instituto Pasteur of São Paulo, Brazil. Her research interests include diagnosis and molecular epidemiology of *Rabies virus*.

### References

1. Pan American Health Organization/World Health Organization. La salud en las Américas, Vol II. Washington: The Organizations; 1998:123-45.

2. Pan American Health Organization/World Health Organization. Vigilancia epidemiológica de la rabia en las Américas 1997. Boletín de Vigilancia Epidemiológica de la Rabia en las Américas 1997, Vol XXIX. Washington: The Organizations; 1997.
3. Alvarez E, Ruiz A. La situación de la rabia en América Latina de 1990 a 1994. Boletín Oficina Sanitaria Panamericana 1995;119:451-6.
4. Morais NB. Wild rabies in Ceará and its implications for public health. Virus: reviews and research. Proceedings of the IXth National Meeting of Virology Vol III/Suppl 1. 22-25 November, 1998. São Lorenzo, Matto Grosso, Brazil.
5. Emmons LH, Feer F. Monkeys (primates). In: Neotropical rainforest mammals, a field guide. 2nd ed. Chicago: University of Chicago Press; 1997. p. 105-45.
6. Mittermeier RA, Coimbra-Filho AF, van Roosmalen MGM. Callitrichids in Brazil and the Guianas: current conservation status and potential for biomedical research. Primate Medicine 1978;10:20-9.
7. Sussman RW, Kinzey WG. The ecological role of the Callitrichidae: a review. Am J Phys Anthropol 1984;64:419-49.
8. de Mattos CC, de Mattos CA, Loza-Rubio E, Aguilar-Setién A, Orciari LA, Smith JS. Molecular characterization of rabies virus isolates from Mexico: implications for transmission dynamics and human risk. Am J Trop Med Hyg 1999;61:587-97.
9. de Mattos CA, de Mattos CC, Smith JS, Miller ET, Papo S, Utrera A, et al. Genetic characterization of rabies field isolates from Venezuela. J Clin Microbiol 1996;34:1553-8.
10. Smith JS, Orciari LA, Yager PA, Seidel HD, Warner CK. Epidemiologic and historical relationships among 87 rabies virus isolates as determined by limited sequence analysis. J Infect Dis 1992;166:296-307.
11. Loza-Rubio E, de Mattos CC, Aguilar-Setién A, de Mattos CA. Aislamiento y caracterización molecular de un virus rábico obtenido de un murciélago no hematófago en la Ciudad de México. Veterinaria México 2000;31:147-52.
12. Diaz AM, Papo S, Rodriguez A, Smith JS. Antigenic analysis of rabies virus isolates from Latin America and the Caribbean. Zentralbl Veterinarmed [B] 1994;41:153-60.
13. Favi CM, Yung PV, Pavletic BC, Ramirez VE, de Mattos CA, de Mattos CC. Rol de los murciélagos insectívoros en la transmisión de la rabia en Chile. Archivos de Medicina Veterinaria 1999;31:157-65.
14. Smith JS. Rabies virus epitopic variation: use in ecologic studies. Adv Virus Res 1989;36:215-53.
15. Conzelman KK, Cos JH, Schneider LG, Thiel HJ. Molecular cloning and complete sequence of the attenuated rabies virus SADB19. Virology 1990;175:485-99.
16. Smith JS. Rabies virus. In: Murray PR, Baron EJ, Pfaller MA, Tenover FC, Yolken R, editors. Manual of clinical microbiology. 6th ed. Washington: American Society for Microbiology Press; 1995. p. 997-1003.
17. Wisconsin Package Version 10.1. Madison, Wisconsin: Genetics Computer Group; 2000.
18. Felsenstein J. PHYLIP Inference Package. Version 3.5c. Seattle: University of Washington; 1993.
19. Page RAM. TREEVIEW: an application to display phylogenetic trees on personal computers. Comput Appl Biosci 1996;12:357-8.
20. Smith JS. New aspects of rabies with emphasis on epidemiology, diagnosis, and prevention of the disease in the United States. Clin Microbiol Rev 1996;9:166-76.
21. Addy PAK. Epidemiology of rabies in Ghana. In: Kuwert E, Merieux C, Koprowski H, Bogel K, editors. Rabies in the Tropics. Heidelberg: Sprnger-Verlag; 1985. p. 497-515.
22. Miot MR, Sikes RK, Silberman MS. Rabies in a chimpanzee. J Am Vet Med Assoc 1973;162:54.
23. Fekadu M. Rabies in Ethiopia. Am J Epidemiol 1982;115:266-73.
24. Rupprecht CE, Smith JS, Fekadu M, Childs JE. The ascension of wildlife rabies: a cause for public health concern or intervention? Emerg Infect Dis 1995;1:107-14.
25. WHO Expert Committee on Rabies. Seventh Report. Geneva: World Health Organization; 1983.
26. Ferrari SF. Ecological differentiation in the Callitrichidae. In: Rylands AB, editor. Marmosets and tamarins: systematics, behavior and ecology. Oxford: Oxford University Press; 1993. p. 314-28.
27. Stevenson MF, Rylands AB. The marmoset, genus *Callithrix*. In: Mittermeier RA, Rylands AB, Coimbra-Filho AF, Fonseca GAB, editors. Ecology and behavior of neotropical primates. Vol. II. Washington: World Wildlife Fund; 1988. P. 131-222.
28. Andrade MR, Nuñes de Oliveira A, Romijin PC, Kimura LS, Costa CC. Infección experimental en primates no humanos (*Callithrix* sp.) con el virus de la rabia: acompañamiento del curso de la enfermedad. Animales de Experimentación, La Revista Hispanoamericana 1999;4:7-10.
